# PCR for Detection of Oseltamivir Resistance Mutation in Influenza A(H7N9) Virus

**DOI:** 10.3201/eid2005.131364

**Published:** 2014-05

**Authors:** Wei Wang, Zhigang Song, Wencai Guan, Yi Liu, Xiaonan Zhang, Lei Xu, Jianhua Li, Zhenghong Yuan, Yunwen Hu

**Affiliations:** Shanghai Public Health Clinical Center, Shanghai, China

**Keywords:** influenza, influenza virus, viruses, H7N9, influenza A(H7N9) virus, neuraminidase, NA 292K mutant virus, R292K wild-type virus, single-nucleotide polymorphism, SNP, real-time reverse transcription PCR, real-time RT-PCR, oseltamivir resistance, mutation

## Abstract

Sensitive molecular techniques are needed for rapid detection of the R292K oseltamivir-resistant mutant of influenza A(H7/N9) virus strain to monitor its transmission and guide antiviral treatment. We developed a real-time reverse transcription PCR and single nucleotide polymorphism probes to differentiate this mutant strain in mixed virus populations in human specimens.

An outbreak of human infections with a novel reassortant avian-origin influenza A(H7N9) virus occurred in several provinces of China during March 2013 (*1*), This outbreak caused 137 laboratory-confirmed cases and 45 deaths as of October 2013 (www.who.int/csr/don/2013_10_24a/en/index.html). An unusually high proportion of severe cases and a high case-fatality rate have been observed for patients infected with this virus (*2*).

We reported emergence of an influenza virus with a mutation in the neuraminidase (NA) gene (R292K) and its association with severe clinical outcome in infected persons (*3*). Studies have shown that the NA R292K mutation can cause a high level of resistance to oseltamivir in influenza A(H7N9) virus (*4**,**5*). Thus, sensitive molecular techniques are needed for rapid detection of influenza virus with this mutation to monitor its circulation and transmission and guide antiviral treatment. In this study, we developed a single-nucleotide polymorphism (SNP) real-time reverse transcription PCR (RT-PCR) to differentiate NA 292K mutant virus from R292 wild-type virus in clinical samples.

## The Study

The NA R292K assay has 2 reactions with 1 pair of primers. One reaction contained a FAM-labeled SNP probe specific for the 292K mutant strain and a second reaction contained a VIC-labeled probe specific for the R292 wild-type strain (Technical Appendix).

To assess the sensitivity of the assay, we constructed 2 plasmids that contained R292 wild-type virus or 292K mutant virus, respectively. Fragments of the NA gene inserted into the plasmids were amplified from nasopharyngeal swab specimens from 2 patients infected with influenza A(H7N9) virus and confirmed by using Sanger sequencing (Technical Appendix). The 2 plasmids were serially diluted 10-fold (10^1^–10^11^ copies) in sterile water and used to test the assay. The linear range of sensitivity was 10^2^–10^8^ copies. The lower limit of detection was 100 copies/reaction (3/3 reactions detected) for wild-type and mutant virus (Figure). However, the sensitivity of the duplex reaction containing both probes was 100-fold lower than that of each separate reaction.

**Figure:**
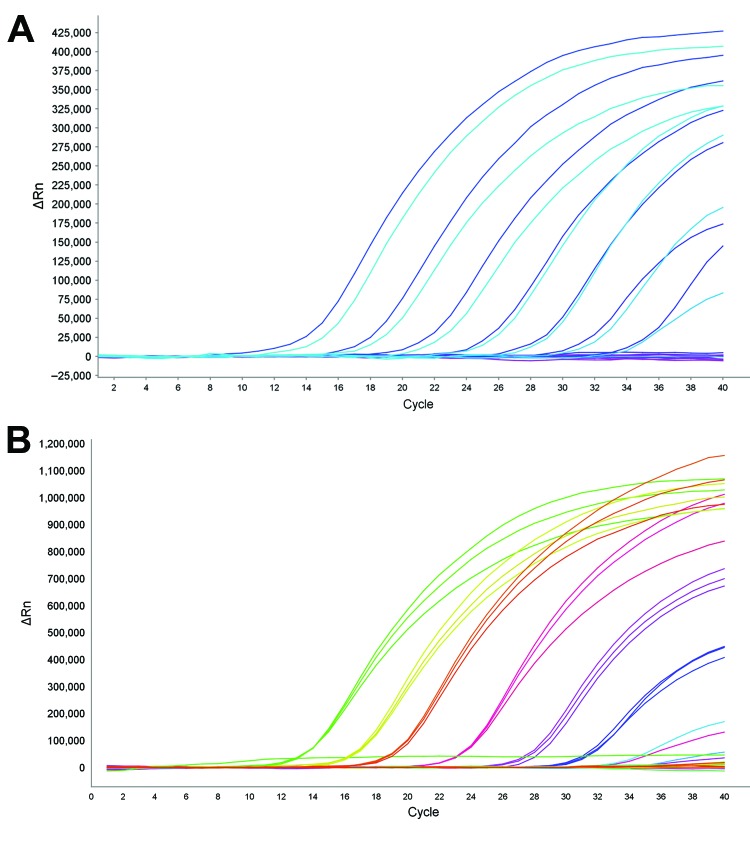
Dynamic range of reverse transcription PCR for detection of oseltamivir resistance in influenza A(H7N9) virus. Amplification curves (ΔRn vs. cycle number) for serial dilutions of plasmid with 292K (mutant) or R292 (wild-type) neuraminidase (NA) fragments. ΔRN is change in signal magnitude (reporter signal minus baseline signal). Assay dynamic range was linear at template concentrations of 10^2^–10^8^ copies/reaction. A) Detection of NA 292K mutant strain with probe N9-K: slope = −3.388, R2 = 0.997. Light and dark blue curves indicate probe NA 292K in duplicate wells. Violet curves indicate control wells. B) Detection of NA R292 wild-type strain with probe N9-R: slope = −3.672, R2 = 0.992. Different colored curves indicate probe N9-R in triplicate wells.

Of 35 respiratory samples tested, 6 were infected with influenza A(H3N2), 2 with influenza A(H1N1) virus, 6 with influenza A(H1N1)pdm09 virus, 4 with parainfluenza virus, 4 with human rhinovirus, 4 with human coronavirus, 5 with influenza B virus, and 4 with respiratory syncytial virus. In addition, 6 other respiratory samples were virus negative. Cross-amplification was not observed during sample testing. Thus, the assay is highly specific for detecting the mutant NA gene of influenza A(H7N9) virus.

To test the performance of the assay when 292K mutant and R292 wild-type viruses were present in 1 sample, a series of mixtures containing the 292K plasmid and the R292 plasmid at copy numbers of 10^4^ copies/reaction were prepared at the following ratios of mutant virus to wild-type virus: 2:98, 5:95, 10:90, 20:80, 30:70, 40:60, 50:50, 60:40, 70:30, 80:20, 90:10, 95:5, and 98:2. The ΔC_t_K – R of the mixture a ratio of 50:50 was used as the assay-specific normalization value in determination of the percentage of 292K mutant in mixed population as described by Liu et al. (*6*). The assay detected the 292K mutant in the mixture at a proportion of 2% of the 10^4^ copies/reaction, and correct estimation of its proportion ranged from 10% to 98% (Technical Appendix).

To validate the assay with clinical samples, we tested 11 paired nasopharyngeal swab specimens and sputum specimens obtained from 9 patients infected with influenza A(H7N9) virus who had various disease outcomes (Table). The time of sampling (mean 12.6 days, range 7–20 days) was at the end of treatment with an NA inhibitor (oseltamivir or peramivir) or afterwards. Eleven of 22 samples were positive for influenza A(H7N9) virus by a quantitative real-time RT-PCR described in a previous study (*3*). Seven of 11 samples had positive results in the R292K assay: 5 samples positive in the 292K assay and 2 samples positive in both assays. Four of 11 samples were negative in both assays. All 292K-positive samples were further confirmed as positive by Sanger sequencing of NA genes.

**Table:** Detection of wild-type neuraminidase R292 and mutant 292K influenza A(H7N9) virus in clinical samples from 9 patients in China, by reverse transcription PCR*

Patient no.†	Outcome	Sample type	Time after oseltamivir treatment started, d	Time after oseltamivir treatment ended, d	292K:R292 ratio	Viral load, copies/mL‡
2	Died	NPS	9	−1	100:0	1.78 × 10^5^
		S	9	−1	100:0	8.76 × 10^4^
		NPS	10	−2	100:0	6.14 × 10^4^
		S	10	−2	25.7:74.3	1.29 × 10^4^
3	Died	NPS	13	3	–	ND
		S	13	3	–	3.03 × 10^3^
		NPS	16	6	–	ND
		S	16	6	100:0	1.21 × 10^4^
5	Died	NPS	7	−4	–	9.96 × 10^2^
		S	11	0	–	ND
6	Recovered	NPS	16	−3	–	ND
		S	19	0	–	ND
7	Recovered	NPS	13	0	–	ND
		S	13	0	–	ND
8	Recovered	NPS	7	0	–	ND
		S	7	0	100:0	1.76 × 10^4^
10	Recovered	NPS	11	−4	–	ND
		S	15	0	91.7:8.3	5.25 × 10^4^
15	Died	NPS	20	4	–	ND
		S	20	4	–	5.62 × 10^3^
17	Recovered	NPS	11	0	–	ND
		S	11	0	–	3.06 × 10^4^

*NPS, nasopharyngeal swab specimen; S, sputum specimen; ­–, no ratio because samples had negative results; ND, not detected. †Patient identification was identical with that used by Hu et al. (*3*). Patients 15 and 17 are initially reported in the current study. ‡Method used to determine viral load was reported by Hu et al. (*3*).

The 7 samples that contained the 292K mutant were obtained from 4 patients: 2 patients who died (patients 2 and 3) and 2 patients who recovered (patients 8 and 10). In our previous study, sequencing of the NA gene was not successful for the first throat swab specimens from patients 8 and 10 because of low viral load (*3*). However, in this study, the 292K mutant was found in sputum specimens from these 2 patients on days 7 or 15, respectively, after initiation of antiviral treatment. This finding suggested that influenza A(H7N9) virus mutated under the pressure of antiviral treatment, which led to failure of the virus to clear the lower respiratory tracts. These 2 patients, who were infected with the drug-resistant mutant virus, recovered from their diseases, which suggested that host immune response might play a major role in controlling the mutant virus.

## Conclusions

Higher viral load in sputum samples indicated that there might be factors, including hemagglutinin (HA) binding preference, which favor greater replication in the lower respiratory tract. A similar phenomenon was observed in patients infected with the HA D222G mutant of influenza A(H1N1)pdm09 virus; this virus showed preferential replication in the lower respiratory tract and this infection was correlated with severe outcomes or deaths (*7*).

We have developed an SNP real-time RT-PCR for detection of a drug-resistant NA R292K mutant of influenza A(H7N9) virus. The sensitivity of the assay is lower than that of an HA7-specific real-time RT-PCR (i.e., 4 samples positive for influenza A(H7N9) virus were not detected by this RT-PCR). However, as a screening tool, this assay is sensitive, specific, fast, and inexpensive.

This assay had a detection threshold of 10% for a mutant strain in a mixed viral population, which is more sensitive than Sanger sequencing (detection threshold of 25% for a minor component in a mixed viral population) (*6*). This assay will help clinicians monitor emergence of drug-resistant virus strains during treatment of patients with NA inhibitors to prevent persistent viral replication and severe inflammatory reactions.

Technical Appendix PCR for detection of oseltamivir resistance in influenza A(H7N9) virus.
