# Supplementary material for: PCR for Detection of Oseltamivir Resistance Mutation in Influenza A(H7N9) Virus
Source: Emerg Infect Dis. 2014 May;20(5):847–9. doi: 10.3201/eid2005.131364 (PMC4012804; doi:10.3201/eid2005.131364)
Supplement: Technical Appendix — PCR for detection of oseltamivir resistance in influenza A(H7N9) virus. [file 13-1364-Techapp-s1.pdf]

Article DOI: http://dx.doi.org/10.3201/eid2005.131364

PCR for Detection of Oseltamivir Resistance in Influenza A(H7N9) Virus

Technical Appendix

Samples and Sample Collection

A total of 11 paired nasopharyngeal swab specimens and sputum specimens were collected from 9 patients who were given a diagnosis of infection with influenza A(H7N9) virus at the Shanghai Public Health Clinical Centre, Shanghai, China, during April 4–27, 2013. Sputum samples treated with 4% sodium hydroxide solution and nasopharyngeal swab specimens were placed in virus transport medium (minimum essential medium containing 2% fetal bovine serum, 5% penicillin/streptomycin, and amphotericin B) immediately after collection and subsequently stored at –80°C. All the samples were detected by using a hemagglutinin 7–specific real-time reverse transcription PCR (RT-PCR) to determine viral load, as described by Hu et al. (*1*).

Plasmid Construction

Template RNA was extracted from nasopharyngeal swab specimens of patients infected with influenza A(H7N9) virus by using the QIAamp Viral RNA Mini Kit (QIAGEN, Hilden, Germany) according to the manufacture’s instructions. Extracted RNAs were amplified by using primers N9-NA346F and N9-NA925R, which are specific for the neuraminidase (NA) gene of influenza A(H7N9) virus (online Technical Appendix Table 1). The PCR products were inserted into the pMD18-T vector (TaKaRa, Dalian, China), and clones were sequenced by using primers N9-NA697F and N9-NA943R (online Technical Appendix Table 1). Clones containing specific fragments with NA R292 or292

K (N2 numbering) were used to generate plasmid standards, which were serially diluted 10-fold (10^1–10^{11} copies) in sterile water and used in assay validation.

Single-Nucleotide Polymorphism Real-Time RT-PCR

Sequences of influenza A(H7N9) viruses from the Global Initiative on Sharing Avian Influenza Data database were aligned by using PrimerExpress software version 3.0 (Applied Biosystems, Foster City, CA, USA). One pair of primers and 2 minor groove–binding probes were designed to discriminate the codon for lysine (AAG) at position 292 in the NA gene for the mutant virus strain and the codon for arginine (AGG) at position 292 in the NA gene for the wild-type virus strain (online Technical Appendix Table 1). The assay was composed of 2 reactions: 1 contained the FAM-labeled probe specific for the 292K mutant strain and 1 contained the VIC-labeled probe specific for the R292 wild-type strain.

Viral RNAs were extracted by using QIAamp Viral RNA Mini Kit (QIAGEN) according to the manufacture's instructions. The reaction was performed by using the One-Step Fluorescent Quantitative RT-PCR Kit (TaKaRA). Real-time RT-PCRs were performed in a 25-μL reaction volume containing 12.5 μL of reaction buffer, 5 μL of viral RNA, 400 nmol/L of each primer, and 200 nmol/L of probe. The reaction program was 42°C for 10 min; 95°C for 30 s; 5 cycles at 95°C for 10 s, 55°C for 20 s, and 72°C for 30 s; and 40 cycles at 95°C for 10 s and 60°C for 40 s. The assay was performed by using the ABI StepOne Plus System (Applied Biosystems), and data were processed by using StepOne software version 2.2 (Applied Biosystems).

Results were considered positive if the cycle threshold (C_t) values were <35 cycles and the ΔRn values (representing changes in the linear amplification plot of the fluorescent signal) were >30,000 for the FAM-labeled probe and 11,000 for the VIC-labeled probe. The baseline and threshold values were set by default. Cross-hybridization was not

observed between the 292R probe and the 292K plasmid or the 292K probe and the 292R plasmid.

Detection of Mixed Population by Using Quantified Plasmid Mixtures

To test the ability of the assay to detect the 292K mutant when both variants are present at various ratios and different viral loads, we prepared mixtures containing the 292K plasmid and the R292 plasmid, respectively, at copy numbers of 10^4 at the following ratios: 2:98, 5:95, 10:90, 20:80, 30:70, 40:60, 50:50, 60:40, 70:30, 80:20, 90:10, 95:5 and 98:2. All mutant:wild-type mixtures were analyzed, and results are shown in online Technical Appendix Table 2. The $\Delta C_t K - R$ value for the mixture at a ratio of 50:50 was used as the assay-specific normalization value in determining the percentage of 292K mutant in mixed populations as dscribed by Liu et al. (*2*). The percentage of the 292K population in the sample was calculated by using the formula of Liu et al. (*2*): $\Delta C_t K - R = C_t K - C_t R - \Delta C_t k50$, where $C_t K$ is the threshold cycle for 292K detection, $C_t R$ is the threshold cycle for R292 detection, and $\Delta C_t k50$ is the $\Delta C_t K - R$ value of the R:K mixture with 10^4 copies at a ratio of 50:50, which was used for normalization. The percentage of 292K on the population was $1/(2^{\Delta C_t k - R} + 1) \times 100$.

Patients 15 and 17

Patient 15 was a 58-year-old man who was admitted to the Shanghai Public Health Clinical Centre on April 7, 2013, because of fever and cough with white sputum for 10 days and polypnea for 5 days. At admission, he had a temperature of 39°C, a pulse rate of 96 beats/min, and a respiration rate of 26 breaths/min. He had hypertension as an underlying condition. Influenza A(H7N9) virus was detected in throat swab specimens on the day of admission. The patient had received oseltamivir, 75 mg twice a day, on April 5. He was then given oseltamivir, 150 mg twice a day, on April 13–15, and peramivir on April 16, He was also given corticosteroids during April 5–26. Extracorporeal membrane

oxygenation was used on June 1 just before the patient died of multiple organ dysfunction. Influenza A (H7N9) virus was weakly detected by quantitative PCR in a throat swab specimen obtained 48 days after admission of the patient.

Patient 17 was a 67-year-old man who was admitted to the Shanghai Public Health Clinical Centre on April 21, 2013, because he had exhibited cough and fever for 8 days. At admission, he had a temperature of 38.5°C, a pulse rate of 76 beats/min, and a respiration rate of 26 breaths/min. Laboratory test showed that he was infected with influenza A(H7N9) virus 1 week before admission. The patient had received oseltamivir, 75 mg twice a day, since April 17. He was then given a double dose of oseltamivir, 150 mg twice a day, during April 21–27. Influenza A(H7N9) virus was not detected in throat swab samples obtained after 15 days of treatment with oseltamivir.. He was considered cured and discharged on May 10.

References

<jrn>1. Hu Y, Lu S, Song Z, Wang W, Hao P, Li J, et al. Association between adverse clinical outcome in human disease caused by novel influenza A H7N9 virus and sustained viral shedding and emergence of antiviral resistance. Lancet. 2013;381:2273–9. PubMed http://dx.doi.org/10.1016/S0140-6736(13)61125-3</jrn>

<jrn>2. Liu CM, Driebe EM, Schupp J, Kelley E, Nguyen JT, McSharry JJ, et al. Rapid quantification of single-nucleotide mutations in mixed influenza A viral populations using allele-specific mixture analysis. J Virol Methods. 2010;163:109–15. PubMed http://dx.doi.org/10.1016/j.jviromet.2009.09.007</jrn>

Technical Appendix Table 1. Primer and probe sequences designed for detection of the NA R292K mutation and clone construction for influenza A(H7N9) virus*

Procedure	Primer or probe	Sequence, 5→3′	Nucleotide location†
NA R292K assay	N9-NA827F	CATGTTACGGGGAACGAACAGG	827–848
	N9-NA894R	TGGTCTATTTGAGCCCTGCCA	874–894
	N9-K	(FAM)-CACATGC**AAG**GACAA-(MGB)	858–872
	N9-R	(VIC)-CACATGC**AGG**GACAA-(MGB)	858–872
Sanger sequencing	N9-NA697F	TGTGTATGCCACAACGGYGTATGCC	697–703
Clone construction	N9-NA346F	GTCACAAGRGARCCTTATGT	346–366
	N9-NA925R	GTGTCATTGCYACTGGRTCTATC	903–925

*NA, neuraminidase. The single-nucleotide polymorphism position is indicated in in **boldface**.

†Numbering is according to the reference sequence A/Shanghai/4664T/2013(H7N9) (GenInfo accession no. KC853231).

Technical Appendix Table 2. Proportion estimation of neuraminidase 292K mutant in influenza A(H7N9) virus mixtures prepared by using quantified R292 and 292K plasmids at copy numbers of 10^4 per reaction*

			% of 292K mutant†	
292K:R292 ratio	C_tK value, mean (SD)	C_tR value, mean (SD)	Mean (SD)	95% CI
2:98	30.4 (1.2)	22.3 (0.1)	0.9 (0.6)	–0.7 to 2.4
5:95	26.8 (0.9)	19.1 (1.5)	0.9 (0.3)	0.1–1.7
10:90	25.4 (0.1)	21.7 (0.4)	12.1 (1.8)	7.6–16.5
20:80	24.7 (0.1)	21.8 (0.2)	20.2 (2.1)	15.0–25.4
30:70	24.6 (0.2)	22.5 (0.2)	29.9 (2.6)	23.6–36.2
40:60	24.1 (0.2)	22.7 (0.1)	40.4 (3.9)	30.9–50.0
50:50	23.7 (0.2)	23.1 (0.1)	55.2 (0.8)	53.3–57.0
60:40	23.9 (0.2)	24.0 (0.2)	67.9 (1.4)	64.4–71.4
70:30	23.6 (0.4)	24.3 (0.3)	74.2 (3.3)	65.9–82.5
80:20	24.0 (0.3)	25.2 (0.3)	80.7 (2.8)	73.7–80.7
90:10	28.7 (0.5)	31.4 (1.3)	91.9 (4.0)	82.0–101.8
95:5	23.6 (0.4)	26.3 (0.7)	92.3 (2.5)	86.3–98.4
98:2	23.4 (0.1)	28.5 (0.1)	98.4 (0.1)	97.3–99.6

*C_tK, cycle threshold for 292K detection; C_tR, cycle threshold for R292 detection.

†Percentage indicates calculated ratio of mutant strain or wild-type strain in mixture population. The method of calculation is as described by Liu et al. (*2*).
